# New diagnostic tools for breast cancer

**DOI:** 10.1007/s12254-017-0341-5

**Published:** 2017-06-28

**Authors:** Pascal A. T. Baltzer, Panagiotis Kapetas, Maria Adele Marino, Paola Clauser

**Affiliations:** Department of Biomedical Imaging and Image-Guided Therapy, Vienna General Hospital, Medical University of Vienna, Währinger Gürtel 18–20, 1090 Vienna, Austria

**Keywords:** Breast cancer, Tomosynthesis, MRI, Elastography, Contrast-enhanced mammography

## Abstract

Imaging plays a major role in the diagnosis, treatment, and follow-up of breast cancer. Findings that require further assessment will be detected both at screening and curative mammography. Most findings that are further worked up tend to yield benign diagnoses. Consequently, there is an ongoing search for new tools to reduce recalls and unnecessary biopsies while maintaining or improving cancer detection rates. The clinically most promising methods in this respect are described and discussed in this review.

## Introduction

Imaging plays a major role in the diagnosis, treatment, and follow-up of breast cancers. While the effect of secondary prevention by means of population-based mammography screening programs is still a matter of debate, the majority of expert societies are clearly in favor of mammographic screening [1]. Although imaging is considered the most efficient diagnostic test for detecting breast cancer, screening mammography does not yield perfect results. In dense breasts, cancers that do not present as mammographic microcalcifications are easily missed and mammographic findings such as masses, architectural distortions, asymmetries, and microcalcifications are not specific for breast cancer. As the aim of imaging is to detect cancer, the reader will regularly choose a diagnostic decision leading to further work-up by additional imaging, follow-up examinations, and image-guided biopsies, leading to extra costs, anxiety, and possible physical harm. Therefore, there is an ongoing search for new diagnostic tools in breast cancer. Key methods include three-dimensional (3D) approaches to x‑ray mammography (digital breast tomosynthesis), contrast-enhanced mammography, quantifiable ultrasound techniques (e. g., shear wave elastography), and functional magnetic resonance imaging (MRI; e. g., contrast-enhanced MRI, diffusion-weighted imaging). This review focuses on these techniques and their potential use in the breast clinic.

## Digital breast tomosynthesis

Digital breast tomosynthesis (DBT) has swiftly gained popularity since its introduction in clinical practice in the 2000s [2]. DBT is a quasi-3D imaging modality that, through the acquisition of a limited number of x‑ray projections from a relatively narrow angular range, allows for the reconstruction of pseudotomographic images [3].

The position of the patient during the examination is identical to that of mammography, and the imaging findings are like those of digital mammography, so that the same diagnostic criteria apply for both modalities. Consequently, the learning curve for becoming accustomed to reading DBT images is rather steep, a fact that has favored the fast increase of its use in clinical practice. While initial DBT devices were associated with a radiation dose approximately twice that of digital mammography, current commercial devices acquire DBT datasets with identical to or only slightly higher doses than mammography [4].

The currently available evidence demonstrates that by use of DBT the detection rate of malignant lesions can be increased. In particular, prospective studies performed in a screening setting showed an increased detection rate from 4.2–6.3/1000 with mammography to 5.4–8.9 with mammography and DBT [5], with a significant increase in detection rates between 0.5 and 2.7/1000 [6]. Retrospective studies showed an increase in sensitivity from borderline (2%) up to 18%, depending on the cases included and the readers’ experience with mammography and DBT [7]. Most of the additional cancers found with DBT, in addition, were invasive rather than in situ carcinomas [8]. Furthermore, false-positive findings leading to unnecessary recalls and biopsies can be reduced. The reduction in recall rates is variable, and is strongly related to the initial recall rate of the screening program where DBT is introduced. In prospective studies in a screening setting, recall rates were stable or decreased by up to 17% [5, 9]. In retrospective studies, specificity improved by up to 20%, with reductions in recall rates of up to approximately 60% [7–10].

In particular, DBT improves the evaluation of soft tissue lesions, while allowing for an adequate evaluation of microcalcifications [11]. Although DBT provides 3D insight into the breast, the community agrees on the necessity of two-dimensional (2D) images to facilitate comparison with previous examinations and the evaluation of microcalcifications. To reduce the radiation exposure from the two examinations (DBT and mammography), the acquired DBT data are used to calculate synthetic 2D mammograms. The synthetic mammogram, despite having a lower image quality than mammography – e. g., due to motion artifacts [12] – allows for a diagnostic performance comparable to that of mammography if read in association with DBT [13, 14]. In a screening setting, the cancer detection rate was above 7.4 per 1000 screenings for DBT with either synthetic 2D mammograms or standard mammograms, with no significant difference [13].

While the acquisition of both standard mammographic views (craniocaudal and mediolateral oblique) is required in the clinical setting [15], the acquisition of a single view has been suggested in the screening setting, yielding promising results [16].

Therefore, DBT has already been suggested as an alternative to mammography in organized population-based breast screening programs. Further analysis, on the other hand, showed that recall rates are not decreased in screening programs with already low recall rates [9, 16]. Data on the effect of DBT on the rate of interval cancers are still limited [17], and thus its implementation in screening programs is still a matter of debate.

## Contrast-enhanced mammography

The ongoing technological development of dual-energy technology facilitated the introduction of contrast-enhanced mammography. As for DBT, images are acquired in the same position in which a standard mammogram is performed.

Before the examination, an iodine-based contrast medium is injected intravenously. Approximately 2 min after the examination, a standard mammography examination is performed. The device acquires two images during the same compression, a low- and a high-energy image. The low-energy image is used as standard mammography, while the high-energy image is post-processed in order to obtain an image in which only the enhancing lesions are visible [18].

The empirical evidence regarding the use of contrast-enhanced mammography is still limited. A meta-analysis of the available data demonstrated high sensitivity (91–100%) but heterogeneous and rather low specificity (32–88%) [19]. In a recent multi-reader analysis of 178 cancer patients [20], the performance of contrast-enhanced mammography was found to be better than mammography alone (area under the curve 0.84 vs. 0.76) and as good as MRI (0.85).

The examination is well tolerated by patients [21] and is of only minor risk to patients when guidelines on the use of iodinated contrast media are followed.

Suggested clinical applications currently overlap with those of breast MRI, ranging from staging, evaluation of inconclusive findings, and even screening of high-risk patients [18, 19]. Despite the initial positive results, the available evidence is not sufficient to recommend a broader use of contrast-enhanced mammography in breast cancer diagnosis and staging if mammography, ultrasound, and MRI are available.

## Acoustic radiation force impulse imaging

Acoustic radiation force impulse (ARFI) is a relatively new development in the field of ultrasound elastography, aiming to evaluate tissue stiffness. A short-duration, high-intensity acoustic “pushing pulse” is transmitted by the probe, which causes subtle displacement of tissue in a perpendicular plane (shear waves). This is then followed by a series of diagnostic intensity pulses used to track these displacements [22]. The velocity of the generated shear waves depends on tissue stiffness and is generally higher in stiffer tissue. ARFI technology enables the qualitative as well as quantitative evaluation of shear wave velocity (SWV) in vivo with the use of either color-coded maps or quantification regions of interest. SWV is measured in meters per second (m/s). Since no manual compression is necessary, this technique has proven to be relatively user-independent and reproducible compared with usual strain elastography [22, 23].

Several studies have shown that ARFI can aid to distinguish benign from malignant breast lesions, using different reconstruction algorithms. Malignant lesions are usually stiffer than benign ones and different, yet quite divergent, cut-off values have been proposed. In our experience, using the most recent reconstruction algorithm (Virtual Touch IQ–VTIQ) and very low precompression (to avoid artificial tissue stiffening), a cut-off value of 3.23 m/s shows a high area under the receiver operating characteristic (ROC) curve of 0.853 [22]. Other studies using VTIQ proposed in part similar, in part different cut-off values [23, 24]. It is evident that more standardization is necessary, especially regarding the degree of precompression applied. Currently a large-scale, multicentric trial is underway to establish generally accepted cut-off values (https://clinicaltrials.gov/ct2/show/NCT02638935).

**Fig. 1:**
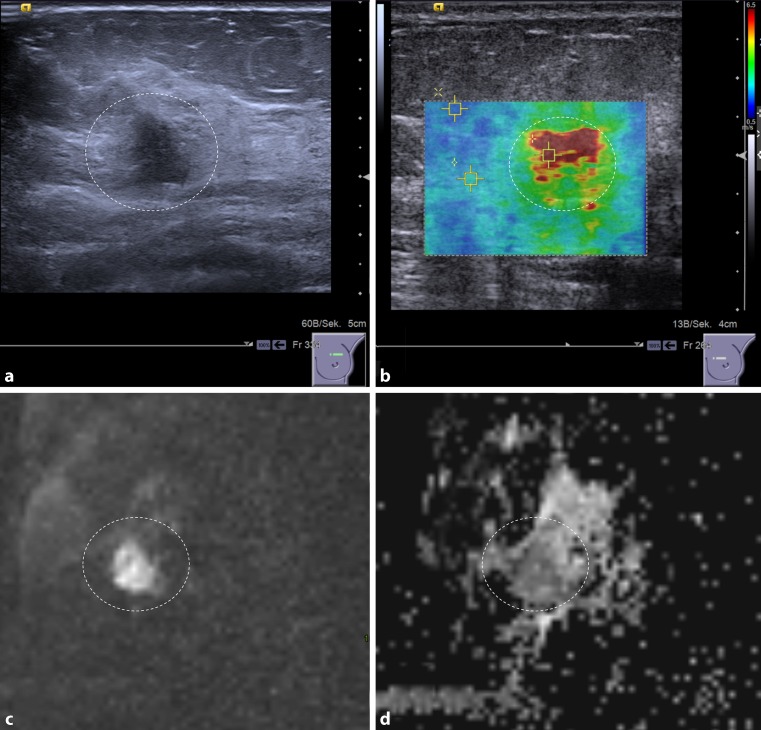
A 48-year-old woman with invasive ductal breast cancer, G3. The lesion (*dashed circle*) presents as an ill-defined hypoechogenic lesion on B‑mode ultrasound (**a**) that is associated with high SWV (4.6 m/s), coded *red* on the parametric ARFI overlay (**b**). The MRI-DWI scan of the same lesion shows a hyperintense lesion (**c**) corresponding to restricted diffusivity (1 * 10^−3^ mm^2^/s) that appears dark on the quantitative ADC map (**d**)

As a potential imaging biomarker, an interesting application of ARFI may be in “ruling in” or “ruling out” malignancy (Fig. 1). Initial data show that it is feasible to downgrade suspicious breast lesions according to B‑mode features without missing any invasive cancers or intermediate-to-high-grade in situ carcinomas if they are associated with a very low SWV. Thus, unnecessary breast biopsies may be avoided in up to 15% of cases [25]. On the other hand, a lesion demonstrating a very high SWV is highly likely to be malignant, irrespective of its B‑mode features and re-biopsy may be indicated in the case of a benign histological result.

Finally, elastography techniques in general have been shown to aid in the early evaluation of response to neoadjuvant chemotherapy with progressive tumor softening in responders. While there is yet no evidence on ARFI in this regard, it can be expected to provide readily available and quantifiable information in this setting.

## Diffusion-weighted magnetic resonance imaging

Contrast-enhanced MRI of the breast is the most sensitive method for detection of breast cancer, in particular of noncalcified lesions [26, 27]. The high sensitivity of contrast-enhanced MRI, however, comes at a price: Many enhancing lesions are not malignant but benign. Interpretation of breast MRI is experience-dependent, and inexperienced readers will in the case of doubt identify a lesion as positive, thus causing unnecessary biopsies and follow-up examinations [28]. To distinguish benign from malignant lesions, a variety of diagnostic criteria can be applied and evidence-based classification rules have been suggested [29]. Incorporating functional perfusion and morphological criteria, this algorithm may improve inexperienced reader performance to the level of experienced radiologists, thereby decreasing inter-reader variation [30]. In addition, the same formal algorithm has the potential to decrease unnecessary biopsies by more than 25% [31]. However, there still is a need for further simple and, if possible, quantitative diagnostic criteria.

Diffusion-weighted imaging (DWI) is an MRI technique that does not require intravenous contrast medium injection. DWI measures water diffusion and thus allows conclusions to be drawn about tissue microstructure [32] (Fig. 1). The microstructural changes that influence water diffusion in neoplastic breast tissue are still poorly understood: In general, neoplastic tissues are characterized by changes in cellularity, proteolytic activity, and reactive desmoplastic induration leading to restricted extracellular water diffusion. DWI sequences qualitatively assess the molecular diffusion, which presents as a high signal when water movements are restricted [32]. Diffusion can be quantified by calculating the apparent diffusion coefficient (ADC) from raw DWI images. High ADC values (>1.4 × 10^−3^ mm/s) correspond to high diffusivity, a finding that generally rules out malignancy. It has been shown that by using a high ADC threshold, unnecessary biopsies may be omitted in different clinical settings [33, 34]. In addition, quantitative ADC measurements used as an imaging biomarker allow invasive breast cancer to be distinguished from in situ breast cancer with a sensitivity of 78% and a specificity of 90% (with a threshold of 1.01 × 10 mm/s) [35]. Finally, quantitative ADC is a promising marker to assess response to neo-adjuvant treatment as it is sensitive to variations in tumor cellularity and necrosis that appear as an increase in ADC values before changes in lesion size and morphology [36].

## Conclusion

**Fig. 2:**
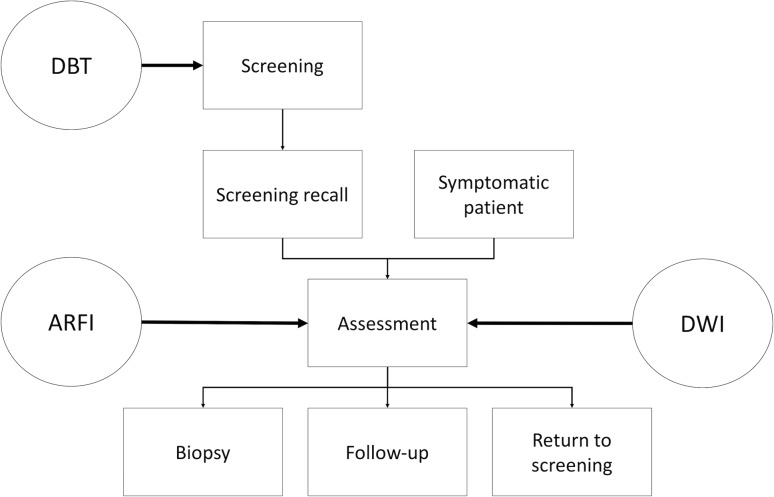
Breast imaging workflow. *Boxes* represent typical steps in the breast imaging workflow, logical steps are connected by *arrows*. *Circles* indicate where the new imaging tools discussed in this article can be of help. *ARFI* acoustic radiation force impulse, *DBT* digital breast tomosynthesis, *DWI* diffusion-weighted imaging

In this short review, we described four new technical developments that aid in breast imaging. While DBT is expected to improve breast cancer screening, ARFI and DWI aid in the assessment of breast lesions (Fig. 2). Contrast-enhanced mammography would potentially fit in, but the necessity of iodinated contrast media, the non-negligible radiation exposure, and limited evidence limit its use as basically all indications would be covered by breast MRI. The potential of all these techniques lies in avoiding unnecessary breast biopsies and in functional assessment of response to pharmacological treatment. Currently, there are still limitations regarding routine application of these techniques: While DBT has not yet shown improved screening outcomes, such improvements are already conceivable considering the evidence so far. Both quantitative techniques (ARFI and DWI) – in particular DWI – have been shown to have diagnostic value; however, there are unresolved issues of standardization that currently preclude the proposal of general thresholds [37]. It is already conceivable that such thresholds need to be established for each center, leading to new challenges regarding quality control procedures [38].
